# Challenges in the Assessment of Bycatch: Postmortem Findings in Harbor Porpoises (*Phocoena phocoena*) Retrieved From Gillnets

**DOI:** 10.1177/0300985820972454

**Published:** 2020-12-04

**Authors:** Lonneke L. IJsseldijk, Meike Scheidat, Marije L. Siemensma, Bram Couperus, Mardik F. Leopold, Maria Morell, Andrea Gröne, Marja J. L. Kik

**Affiliations:** 1 Division of Pathology, Department of Biomolecular Health Sciences, 90051 Faculty of Veterinary Medicine, Utrecht University, The Netherlands; 2 90045 Wageningen Marine Research, Ijmuiden, the Netherlands; 3 Marine Science & Communication, Driebergen-Rijsenburg, the Netherlands; 4 90045 Wageningen Marine Research, Den Helder, the Netherlands; 5 Institute for Terrestrial and Aquatic Wildlife Research, University of Veterinary Medicine Hannover, Büsum, Germany

**Keywords:** diagnostics, incidental capture, bycatch, gillnets, North Sea, postmortem investigation, pathology, *Phocoena phocoena*

## Abstract

Bycatch is considered one of the most significant threats affecting cetaceans worldwide. In the North Sea, bottom-set gillnets are a specific risk for harbor porpoises (*Phocoena phocoena*). Methods to estimate bycatch rates include on-board observers, remote electronic monitoring, and fishermen voluntarily reporting; none of these are systematically conducted. Additionally, necropsies of stranded animals can provide insights into bycatch occurrence and health status of individuals. There are, however, uncertainties when it comes to the assessment of bycatch in stranded animals, mainly due to the lack of diagnostic tools specific for underwater entrapment. We conducted a literature review to establish criteria that aid in the assessment of bycatch in small cetaceans, and we tested which of these criteria applied to harbor porpoises retrieved from gillnets in the Netherlands (*n* = 12). Twenty-five criteria were gathered from literature. Of these, “superficial incisions,” “encircling imprints,” and “recent ingestion of prey” were observed in the vast majority of our confirmed bycatch cases. Criteria like “pulmonary edema,” “pulmonary emphysema,” and “organ congestion” were also frequently observed, although considered unspecific as an indicator of bycatch. Notably, previously mentioned criteria as “favorable health status,” “absence of disease,” or “good nutritional condition” did not apply to the majority of our bycaught porpoises. This may reflect an overall reduced fitness of harbor porpoises inhabiting the southern North Sea or a higher chance of a debilitated porpoise being bycaught, and could result in an underestimation of bycatch rates when assessing stranded animals.

The incidental or unintended capture of cetaceans in fishing nets, hereafter referred to as “bycatch,” is considered one of the most significant threats to marine mammals worldwide.^7,22,40,53^ In the North Sea, this problem is particularly related to bottom-set gillnets.^66^ Here, the harbor porpoise (*Phocoena phocoena*) is the most frequently encountered cetacean, both in terms of sightings and strandings.^14,20^ Harbor porpoises in the North Sea are protected under the EU Habitats Directive and the Agreement on the Conservation of Small Cetaceans of the Baltic and North Seas (ASCOBANS). The latter requirement aims to reduce overall human-induced mortality to below 1.7% of the best population estimate, and specifically to reduce bycatch below 1%.^4,57^ Assessment of bycatch in commercial bottom-set gillnets in the Greater North Sea indicated that between 1175 and 2126 porpoises are bycaught annually (0.33% to 0.59% of the population); however, the authors highlighted that the underlying effort data were incomplete, which likely led to an underestimation of bycatch rates.^18^ Bycatch mortality remains one of the major concerns that may negatively affect harbor porpoise populations.^7,21,52,60^

There are different methods to estimate bycatch rates and calculate the total number of bycaught marine mammals per fishing meter, area, and time. These include on-board observers, remote electronic monitoring (REM), as well as fishermen voluntarily reporting bycatches.^3,27,52,66^ Collecting representative and reliable data for the relevant fishing fleets has been challenging. While it is known that the primary fishing gear causing bycatch in harbor porpoises and other small cetaceans are bottom-set gillnets,^53,66^ the coverage with observer programs in this (and many other types of) fisheries has been proven inadequate to result in management action.^7^ Information on bycatch occurrence can alternatively be gained indirectly from postmortem examinations of stranded animals. Fishermen generally discard bycaught cetaceans.^29^ Depending on environmental factors like wind and tides, a percentage of discarded porpoises will eventually make landfall.

There are uncertainties when it comes to the assessment of bycatch in stranded animals and the biggest challenge is the diagnosis of drowning following underwater entrapment.^39^ In 2002, drowning was defined by an international expert committee of the World Health Organization as “the process of experiencing respiratory impairment from submersion/immersion in liquid” with outcomes being “death, morbidity and no morbidity.”^65^ Death by drowning may occur when fluid is aspirated, resulting in respiratory obstruction and asphyxia, and changes secondary to fluid aspiration. However, aspiration of fluid does not always occur in submersed animals, as death due to laryngospasm can result from the combined effects of asphyxia.^65^ Drowned cetaceans do not frequently have grossly apparent or voluminous seawater within their lungs. This has been attributed to their diving adaptations.^11,23,28^ The eventual cause of death in submerged marine mammals is therefore most often hypoxia. Hypoxia and subsequent asphyxiation cause gross and histological changes to the heart and lungs, among other organs.^23,62^ However, the presence of these changes in dead, stranded cetaceans may not solely be caused by drowning due to underwater entanglement. This highlights the need to assess further criteria that aid in the diagnosis of drowning following bycatch.

Throughout the past decades, several studies have established criteria for the diagnosis of drowning following bycatch in cetaceans, and the diagnosis of “death by submersion” is based on the presence or absence of a number of characteristics.^1,24,29^ However, pathological findings in bycaught individuals may vary depending on local fishery practices and gear used, but also anatomical features of species involved, and therefore the pathologic indicators of bycatch may differ geographically.^11^ In this study, we first conducted a literature review to establish criteria that aid in diagnosing underwater entrapment in small cetaceans, and second, we tested which of these criteria can be applied on confirmed bycaught harbor porpoises that were retrieved from gillnets in the Netherlands. The ultimate aim was to identify which criteria aid in the assessment of bycatch in small cetaceans from the southern North Sea area and should be used to assess the prevalence of bycatch among stranded individuals.

## Materials and Methods

### Review of Bycatch Criteria

The “Proceedings of the Second European Cetacean Society Workshop on Cetacean Pathology: Diagnosis of By-catch in Cetaceans” were used to establish the baseline of bycatch criteria for deceased small cetaceans.^29^ In addition, a literature search was conducted, and we selected studies after 1996 that presented postmortem results of bycaught small cetaceans (delphinids or phocoenids), which were directly retrieved from gillnets. These publications were reviewed for descriptions of postmortem findings in bycaught animals, or for criteria for which these animals were assessed, and these findings were tabulated. Criteria listed in the *Diseases of Aquatic Organisms* theme section on “Criteria and Case Definitions for Serious Injury and Death of Pinnipeds and Cetaceans Caused by Anthropogenic Trauma” were additionally included.^42^ Publications that solely described postmortem findings in stranded animals or those describing postmortem findings from bycaught animals in other types of fisheries were not included.

### Bycaught Harbor Porpoises

Since 2008, postmortem examinations on marine mammals are conducted at the Faculty of Veterinary Medicine, Utrecht University in the Netherlands. Harbor porpoises included in this study were all bycaught animals that were directly obtained from fishing nets or fishermen (referred to as “confirmed bycaught harbor porpoises”). Between 2008 and 2019, 4 harbor porpoises were available for examination following entanglement in gillnets. Additionally, from June 2013 to June 2017, 14 Dutch commercial bottom-set gillnet fishing vessels were equipped with closed-circuit television cameras to assess bycatch rates.^56^ During this project, 8 bycaught harbor porpoises were brought ashore for postmortem examination. All harbor porpoises were caught in bottom-set single-walled gillnets and trammel nets in Dutch coastal waters (Supplementary Fig. S1).

### Necropsies of Bycaught Animals

The necropsies were conducted following internationally standardized guidelines.^19^ The following data were collected from all cases: date and location, sex, total body length (measured from the tip of the rostrum to the fluke notch, in a straight line next to the body, in cm), and mass (in kg). All animals were given a decomposition condition code (DCC), from DCC1 representing very fresh carcasses to DCC4 representing severely autolyzed carcasses. The nutritional status was visually assessed based on the dorsal musculature, presence of visceral fat and blubber thickness. The latter was measured immediately anterior to the dorsal fin at 3 locations (dorsal, lateral, and ventral, in mm). A nutritional condition code (NCC) was appointed on a 6-point scale with 1 representing very fat and muscular animals and 6 representing emaciated animals. Based on total length, animals were assigned an age class, with animals <91 cm classified as neonates, 91 cm to 130 cm considered juvenile, and >130 cm classified as adults. Age was estimated for animals <120 cm as either 1 or <1 depending on the month it was caught, with May as cutoff month, and determined for cases >120 cm following earlier published methods.^17^

The organs of all cases were examined grossly. Parasite presence and severity were scored for 4 major organs (lung, liver, stomach, and middle ear/sinuses) as none, mild, moderate, and severe.^59,63^ From the animals which were in DCC 1 to 2 at the time of necropsy (*n* = 10), a range of tissue samples were collected for histopathology, fixed in 10% neutral buffered formalin, embedded in paraffin, sectioned at 4 to 7 μm, stained with hematoxylin and eosin (HE), and microscopically examined. Photographs of external features taken during necropsies, and gross and histopathological reports were retrospectively assessed for the criteria listed following the literature review. In addition, from cases for which paraffin-embedded tissues were available, skeletal muscle was stained with phosphotungstic acid-hematoxylin (PTAH),^49^ to assess striated muscle fibers (*n* = 8) and lung tissue was stained with Gomori silver,^28^ to reveal any damage to the reticulate fibers of the lungs (*n* = 9). Control tissues of a harbor porpoise which was euthanized following live stranding were also stained with PTAH and Gomori silver.

### Diet Analysis

Prey remains from stomachs were examined following earlier published methods.^33^ Briefly, first and foremost fish sagittal otoliths were used to identify fish species and to estimate fish length and weight. Prey mass was back-calculated.^34^ A total of 9 different prey species were found and these were subsequently grouped into 2 prey guilds: demersal and pelagic.^35,36^ The percentage of demersal prey mass (in grams) out of the total reconstructed prey mass was calculated for each porpoise.

### Inner Ear Analysis

The 4 inner ears of 2 very fresh individuals (sampled and fixed within 18 hours postmortem) were collected following earlier published methods.^43^ The ears were transported to the University of British Columbia (UBC), Canada, for analysis, with appropriate CITES permits (14NL220354/12, 14CA01694/CWHQ-1, 16NL231424/12, and 16CA02279/CHWQ). The inner ears were processed for scanning electron microscopy (SEM) and histopathology in the first individual, and for SEM and immunofluorescence for the second individual, following previously optimized protocols.^44–47^ The sensory cells of the organ of Corti were labelled with anti-prestin (Santa Cruz, SC-22692, 1:200) and anti-myosin VI (Proteus Biosciences, 25-6791, 1:500) antibodies and type I afferent innervation was labelled with anti-neurofilament 200KD (Sigma-Aldrich N0142, 1:400). Nuclei were counterstained with DAPI (4′,6-diamidino-2′-phenylindole, dihydrochloride; Thermo Scientific, 62247). The inner ears were observed using an S-4700 SEM and an Olympus FV1000 confocal microscope at the UBC Bioimaging Facility.

## Results

### Review of Bycatch Criteria

A total of 25 criteria that may aid in the assessment of bycatch were retrieved from the literature. The criteria were divided into 4 main topics: findings related to the drowning process, findings related to contact with or hauling of the net, findings related to disentanglement of bycaught animals, and findings related to the health status of the bycaught individuals (Table 1; references in Supplemental Table S1).

**Table 1.** Bycatch Criteria Retrieved From the Literature^a^.

Criteria	Description
*1. Findings related to the drowning process*	
Hyphema	Hemorrhage in the anterior chamber of the eye, with the eye grossly appearing as red, bulging, and bloody
Pulmonary edema	Blood-tinged or white foam, or fine persistent froth in trachea or elsewhere in the respiratory tract (sometimes protruding from blowhole). Lungs may be heavy and voluminous.
Pulmonary emphysema	Focal or generalized emphysema, such as large air cavities in the lung parenchyma
Pleural petechiae or pulmonary hemorrhage	Pinpoint hemorrhage in the lung pleura or in the lung parenchyma
Changes in reticulum fiber structures of lung	Histological changes such as distention and discontinuity of reticular fibers (confirmed using Gomori silver stain or comparable)
Regurgitation of food	Presence of partly digested stomach content in the upper alimentary tract
Epicardial petechiae	Pinpoint hemorrhage in the epicardium
Organ congestion	Diffuse reddening and/or swelling of adrenal, brain, heart, kidneys, liver, lungs, or spleen
Disseminated congestion	Congestion of 2 or more organs as listed above
Presence of foreign material in lungs or bones	For example, marine flora and fauna (diatoms), microscopically visible in airways
*2: Findings related to contact with or hauling of the net*	
Superficial incisions in edges of the mouth, fins, or tail (stock)/fluke	Incisions (nicks and notches), mostly superficial but penetrating the epidermis, sometimes also in the underlying dermal tissue. There may be underlying hemorrhage, but this is not required. In lip cuts, a tooth can be missing. Morphologically, there should be no signs of chronic tissue response (eg, inflammation or fibrosis), and histopathological assessment of lesions is needed for confirmation.
Encircling lesions, anywhere on the body	Sharp-edged line imprints, mostly around the rostrum, head or around extremities, sometimes resulting in incisions in the edges of tissues (see above)
Subcutaneous hemorrhage/contusions	Hemorrhage in the subcutaneous tissue, such as on the skull (mainly peri-mandibular) or peri-scapular
Intramuscular hemorrhage, myofiber degeneration, or skeletal muscle contusions	Hemorrhage in epaxial and hypaxial muscles, and in thoracic rete mirabile. Histological changes include myofiber degeneration or myopathy (confirmed using PTAH or a comparable stain).
Disseminated gas bubbles	Intravascular and interstitial gas bubbles (from hauling out deceased animals with nitrogen-saturated tissues). Diagnosis depends also on histologic absence of bacteria and autolytic changes.
Pneumothorax	Partial or complete collapse of the lung(s), with air- or gas-filled space in the thorax
(Acute) skull or rib fractures	May or may not have associated hemorrhages (fractures can occur after death when the net is hauled)
*3: Findings related to disentanglement of bycaught animals*	
Amputations	Straight (sharp-edged) removal of extremities (mostly the tail/fluke, with cut through the intervertebral disc; typically cut off after death to release animal from the net)
Presence of fishing material around body extremities	Presence of fishing gear such as rope or monofilament netting materials on the animal
Gaff marks	Angular mutilation(s), could be anywhere on the body (typically incurred after death to move carcasses aboard)
Penetrating incision wounds or lacerations into body cavities	Postmortem cuts (tissue tears) into the body cavities to release gas, possibly induced when releasing an animal from the net or to make a carcass sink
*4: General health status of bycaught animals*	
Good nutritional condition	Well-developed musculature, thick blubber-layer, visceral fat may be present
Low incidence of parasitism	Absence or low incidence of parasites in respiratory and gastrointestinal tracts, liver, and middle ear, among other organs
Exclusion of alternative causes of death	Absence of other, significant gross or histologic lesions to explain death
Recent ingestion of prey	Stomach or upper alimentary tract contains (at least some) undigested or whole prey

^a^ For references^1,2,12,24,28,29,32,37,41,58,59,64^, see Supplemental Table S1.

### Postmortem Results of the Bycaught Harbor Porpoises

Of the 12 bycaught porpoises, 10 were juveniles ranging in size from 93.5 to 117 cm (7 males and 3 females). The other 2 harbor porpoises were adults; a 10-year-old pregnant female of 141 cm, and an 8-year-old resting female of 171 cm. All 12 animals were in a very fresh or fresh condition (DCC1-2) at the time of retrieval from the net, with the exception of an adult resting female that had a DCC4. One very fresh animal was temporarily frozen at −20 °C prior to further examination (for full details on all cases, see Supplemental Tables S2, S3).

#### Findings Related to Drowning Process

Hyphema could be observed grossly in 11 porpoises and was unilateral in 3 cases and bilateral in 2 cases (in total: 5/11). Pulmonary edema, where the alveoli were filled with pale eosinophilic liquid, was both grossly and histologically visible in 10/10 cases in which it could be assessed. Pulmonary emphysema was histologically detected in 8/10 cases, and pleural petechiae or pulmonary hemorrhage in 3/10 cases in which it could be assessed. Digested food remains in the upper alimentary tract (indicative of regurgitation) were observed in 4/11 cases. Epicardial petechiae were histologically observed in 2/10 cases. Congestion was histologically observed in one or more organs for 10/10 cases, mostly in the lungs (9/10), adrenal and kidneys (both 8/10). Additionally, congestion was present in brain (6/9), heart and spleen (both 5/10), and liver (4/10). One case had congestion solely in the lung. All other cases had disseminated congestion affecting from 2 organs to all organs. No foreign material was observed grossly or histologically in lung tissue of any case. Changes in reticulum fiber structures of the lungs were deemed unspecific, with the Gomori silver stain not adding information in addition to that observed in HE-stained sections (Supplemental Table S3).

#### Findings Related to Contact With or Hauling of the Net

**Figures 1–6.**
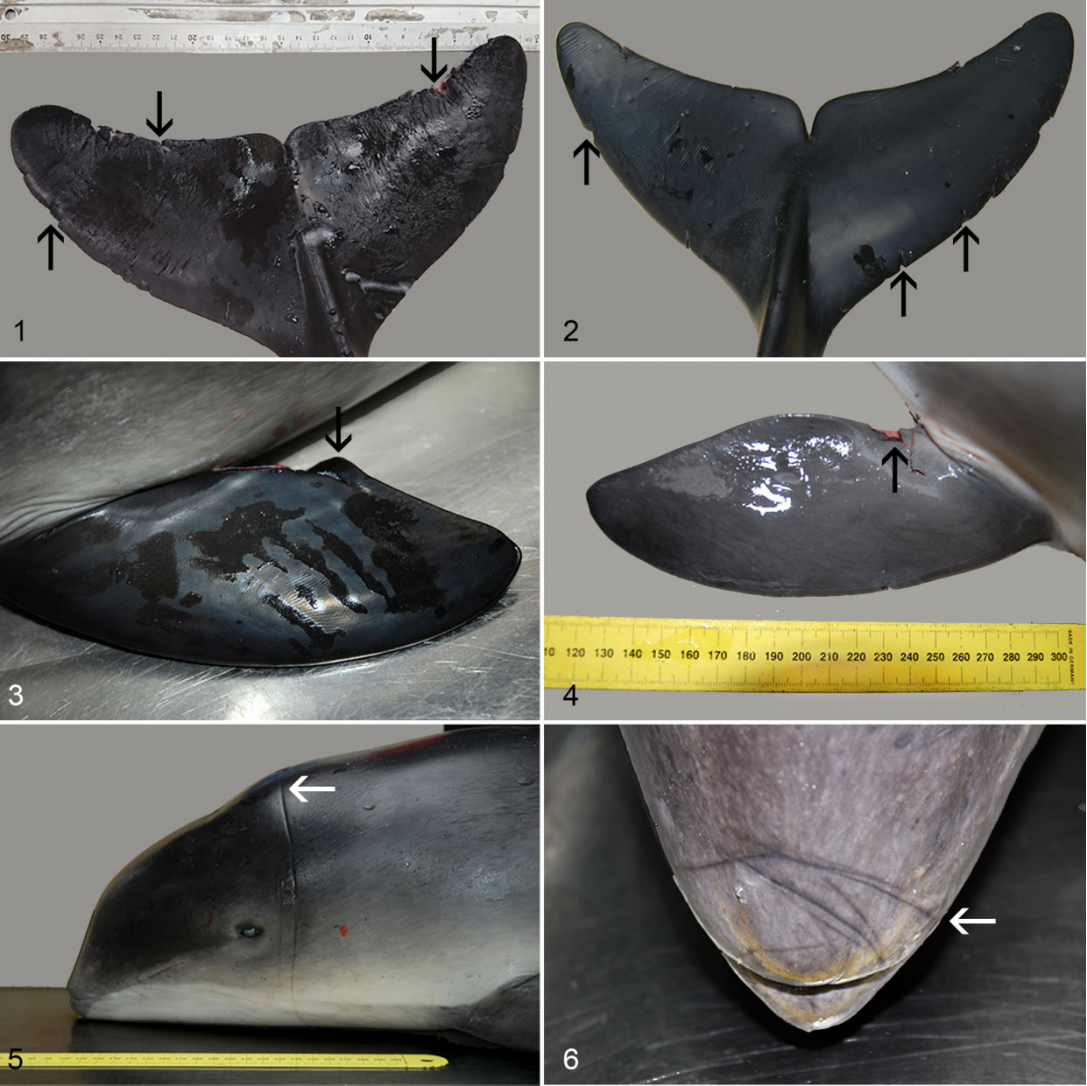
Netmarks and imprints as a result of entanglement, harbor porpoises. **Figures 1–2.** Fluke, cases 12 and 2. Incisions in the edges of the flukes (arrows). **Figure 3.** Left pectoral fin, case 11. Loose-hanging skin tissue (arrow) on the fin. **Figure 4.** Right pectoral fin, case 5. Incision and loss of epidermis (arrow) on the fin. **Figure 5.** Head, case 3. Encircling imprints around the head (arrow). **Figure 6**. Rostrum, case 8. Encircling imprints around the rostrum (arrow).

Incisions were present on the extremities of 12/12 cases: on the pectoral fins (*n* = 12), dorsal fin and fluke (*n* = 10), and on the edges of the maxilla and/or mandible (*n* = 7). Grossly, the severity and extensiveness of these incisions varied between animals (Supplemental Table S4; Figs. 1–6). In the DCC4 case (case 9), imprints were apparent on the dorsal fin, pectoral fins, and fluke, although sloughing of the epidermis as a result of postmortem changes was also apparent. Histologically, lesions showed focal loss of epidermis in all cases assessed (*n* = 9) with evidence of a tissue response in 6 cases that mostly consisted of a mild amount of hemorrhage (Supplemental Table S3). Encircling imprints were grossly present in 8/12 cases, on the rostrum (*n* = 6) or head (*n* = 2). Subcutaneous hemorrhage was observed in 4/12 cases, at a different location in each animal: caudodorsal skull; mandible or submandibular tissue, head, and scapula. Myofiber degeneration was mild and only observed in one of the skeletal muscles (*M. longissimus dorsi*) stained with PTAH. Striations in the control muscle of the live-stranded and euthanized harbor porpoise stained PTAH-positive. One animal had an acute, ante-mortem unilateral fracture of the mandible. Grossly, there were no prominent amounts of gas bubbles noted in any of the cases. Retrospective histologic assessment of liver, spleen, heart, and lymph nodes revealed only one animal with very few gas bubbles in the prescapular lymph node. None of the animals had any evidence of pneumothorax (Supplemental Table S3).

#### Findings Related to Disentanglement

None of the animals had any fishing material still present on arrival to the faculty. None presented with gaff marks, amputations, penetrating incision wounds, or lacerations into the thoracic or abdominal cavity (Supplemental Table S3).

#### Health Status of Bycaught Individuals

**Figure 7.**
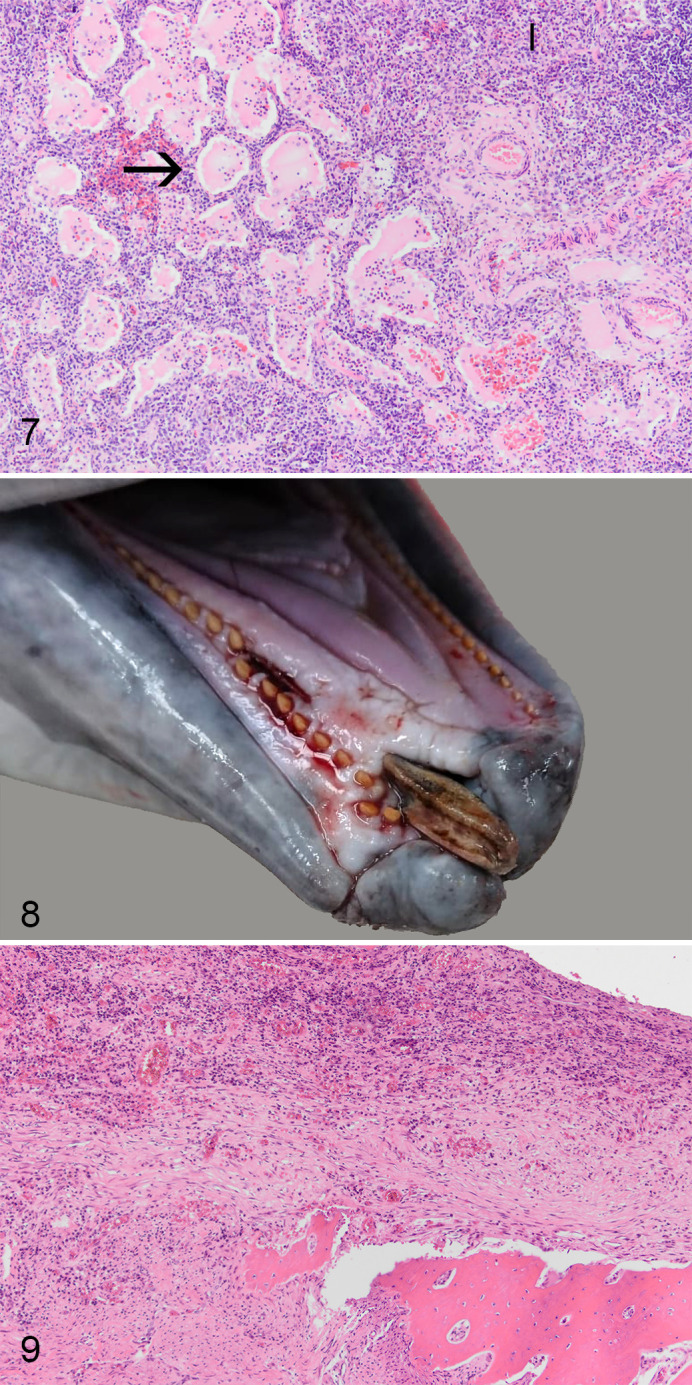
Pneumonia and alveolar edema, lung, harbor porpoise, case 6. There is alveolar edema (arrow) and interstitial infiltrates (I) of leukocytes. Hematoxylin and eosin. **Figures 8–9.** Mandibular osteomyelitis, harbor porpoise, case 12. **Figure 8.** Displacement of the right mandibular bone. **Figure 9.** There is loss of bone tissue, fibrosis, and infiltration of lymphocytes and neutrophils. Hematoxylin and eosin.

Five animals were in good nutritional condition (NCC1–2), 5 were in moderate nutritional condition (NCC3–4), and 2 were emaciated (NCC6; Supplemental Table S2). Parasitic infestation was higher in lungs and middle ears compared to stomach and liver, but was generally mild to moderate (Table 2). Verminous bronchopneumonia was diagnosed in 9 cases, with a suspected bacterial coinfection (based on histopathology) in 4 cases (Fig. 7). Other significant morphological changes included moderate to severe dermatitis in 4 cases, chronic hepatitis in 2 cases, and 1 case each of encephalitis, myocarditis, and mandibular osteomyelitis following a previous trauma (Figs. 8, 9). No ancillary testing was conducted to establish the causes of detected infections. No significant lesions were observed in the one animal which was frozen prior to the necropsy; however, freezing artefacts may have hampered the diagnosis of microscopic lesions.

**Table 2.** Number of Cases With Parasite Infestation in the Lungs, Stomach, Liver, and Middle Ears of 12 Confirmed Bycaught Harbor Porpoises.

Parasite infestation	None	Mild	Moderate	Severe
Lungs	3	4	5	0
Stomach	10	0	1	1
Liver	11	1	0	0
Ears	2	2	2	4

The SEM analysis of one of the inner ears revealed severe hemorrhage due to parasite migration into the right cochlea, suggestive of impaired hearing prior to death (details previously published^45^), and an additional focal hemorrhage in one cochlea of the other individual.

The stomach of the DCC4 adult female was empty. The stomachs of the other 11 cases were assessed for food remains, revealing an overall percentage of demersal prey of 98%. The number of prey items varied, with a minimum of *n* = 15 prey items and a maximum of *n* = 1679 (prey species composition, total prey numbers, and reconstructed masses are given in Supplemental Table S5).

Based on the state of decomposition, it was believed that one of the emaciated animals was bycaught when already dead. When excluding this case, in 4 of 11 bycaught harbor porpoises, no significant gross or histologic lesions could be detected that could have resulted in debilitation of the animals and therefore these were considered healthy at the time they were bycaught. However, the other 7 animals had signs of disease or debilitation, including 4 with severe or extensive morphological changes that likely had a negative effect on these animals.

## Discussion

A total of 25 criteria that aid in the assessment of bycatch in small cetaceans were gained from literature (Table 1, Supplemental Table S1). Twelve harbor porpoises that were bycaught in gillnet fisheries in Dutch coastal waters were retrospectively assessed for the presence of these conditions. Eight criteria were evident in at least two thirds of these confirmed bycatch cases: pulmonary edema, pulmonary emphysema, single-organ congestion, disseminated congestion, presence of superficial incisions on extremities, presence of encircling imprints, low incidence of parasites, and recent ingestion of prey. The other 17 criteria were diagnosed less frequently. Results are discussed by topic below with a focus on the applicability of the bycatch criteria in stranded harbor porpoises.

### Findings Related to the Drowning Process

Pulmonary changes and congestion of organs were present in the majority of the bycaught porpoises, but are not by themselves indicative of bycatch or drowning. Lung edema (where the alveoli are filled with pale eosinophilic liquid) is also frequently observed in live stranded cetaceans and in animals dying of predatory attacks.^13^ It has also been suggested that lung edema develops after death due to intrinsic contractions resulting from activity of the atrioventricular and sinoatrial nodes in the heart.^8,24^ Pulmonary emphysema also occurs in association with pulmonary disease. Pneumonia is one of the most common diagnoses in stranded harbor porpoises^23,60^ and was detected in varying severity in 9 animals in this study (Supplemental Table S3). Organ congestion may also occur following euthanasia,^24^ or from various physiological processes.^48^ A study on drowned and nondrowned dogs concluded that pulmonary congestion, edema, and hemorrhages in the lung were present in both groups and therefore was deemed an unreliable indicator of drowning.^50^ In addition, changes in reticulum fiber structures in lung tissues as a histological criteria for death by drowning^28,53^ could not be confirmed in later investigations,^15^ nor in this study.

Hyphema in cetaceans was significantly related to drowning by others^1,13^ and was observed in 5 of the 11 confirmed bycatch cases. It presumably results from systemic hypertension, which may occur as result of hypoxemia during drowning. However, it cannot be ruled out that this was a result of blunt force trauma,^62^ as 2 of the porpoises with hyphema also had subcutaneous hemorrhage on the head (Supplemental Table S3). As severe and prolonged physical struggle while being trapped in gillnets is not expected (see below), there is a possibility that blunt force trauma occurred later such as when landing the net onboard the fishing vessels when animals were still alive. Thus, hyphema might also be a criterion under “findings related to contact with or hauling of the net.” Subconjunctival hemorrhages have been described in animals stranded in association with acoustic disturbances,^9^ following canid^1^ or grey seal attacks,^13^ or ocular blunt trauma,^54^ and may occur following altered intrathoracic or intraabdominal pressure to veins and capillaries of the head and neck region (eg, due to vomiting, parturition, coughing).^6^ Hyphema as a single criterion can therefore be considered unspecific in bycatch assessment, although informative on the diagnosis of a number of causes. Importantly, the absence of this criterion does not rule out drowning due to bycatch, as more than half of our cases did not have hyphema.

Other criteria related to the drowning process were seen in less than one third to none of the bycaught harbor porpoises, including pleural petechiae or hemorrhage, epicardial petechiae, regurgitation of food, and presence of foreign material in lungs. Assessment of these criteria in stranded harbor porpoises is therefore deemed uninformative in order to establish bycatch rates. It is worthy to note, however, that in human forensic pathology, the diatom test is considered by some as the “gold standard” for the diagnosis of drowning,^51^ although the utility and reliability of this method is considered controversial by others.^38^ In our study, no sign of foreign material was observed in histological sections of lung, although no further attempt was made to specifically assess the presence of diatoms. This method has proven to be informative on the diagnosis of drowning in dogs^50^ and terrestrial wildlife,^10^ but less useful in animals that live in the marine environment and have fish in their diet, like otters,^10^ cetaceans, and sea turtles.^55^ Its applicability for marine species therefore requires further investigation.

### Findings Related to Contact With or Hauling of the Net

Superficial incisions in extremities and encircling imprints were present on all of the bycaught porpoises and therefore deemed most informative in the assessment of bycatch. All lesions were of an acute nature. Detection of such lesions is, however, strongly influenced by postmortem changes. Decomposition results in sloughing of skin, as also observed in the DCC4 case in this study, and additionally scavengers may quickly mask external features. When bycaught marine mammals are discarded and eventually strand, they may do so on mussel beds or rocky shores, which could damage the epidermis and underlying tissue, masking the important external features that reflect entanglement. Additionally, transport of carcasses in body bags and subsequent storage prior to postmortem examination, such as in freezers or chillers, could also leave imprints.^24^ When assessing bycatch in stranded individuals, the likelihood of the occurrence of nicks and notches from other origins that might mimic net imprints should therefore always be considered. It is therefore strongly recommended to take photographs of a carcass and its (stranding) location immediately when it is found, but at least prior to handling the carcass.

Mild myofiber degeneration with minimal loss of visible cross-striation was observed in skeletal muscle of one case. The myofiber degeneration and necrosis with loss of cross-striation was more extensive in the live-stranded and euthanized harbor porpoise (control case). The period between capture and death following submersion is likely in the order of minutes,^61^ leaving limited time for animals to overexert, hence explaining the lack of myofiber degeneration. Subcutaneous or intramuscular hemorrhages were also not frequently detected, indicating a lack of severe physical struggle while trapped.^26^

Other criteria listed under this topic, including fractures, pneumothorax, and gas bubbles, were seen infrequently or not at all in the bycaught harbor porpoises. In previous studies, the presence of gas bubbles was significantly higher in bycaught marine mammals compared to stranded animals.^1,41^ Gas bubbles might be induced following hauling of the net, by bacterial and autolytic changes, or could result from dissection artefacts.^25,41^ Only one of the bycaught harbor porpoises (#2, a very fresh carcass [DCC1] at the time of the necropsy) had a minor amount of gas bubbles in a lymph node, which could have been a result of hauling of the net in which it was bycaught. However, the absence of this finding in the majority of our cases is most likely explained by a difference in the depth at which the gillnets were set compared to previous studies.^1,41^ Bycaught cetaceans with evidence of bubbles in the veins and/or tissues came from gillnets set at a depth of around 80 m,^41^ while the porpoises included in this study were retrieved from bottom-set single-walled gillnets and trammel nets set in coastal waters of less than 20 m deep. Gas bubbles may therefore only form after hauling of nets from greater depths.

### Findings Related to Disentanglement

Criteria listed under this topic included presence of fishing material, amputations, penetrating wounds, and gaff marks. These were diagnosed in none or less than a third of the bycaught harbor porpoises, indicating that these criteria may not be useful for detecting fisheries-related mortality in stranded animals. However, many of these bycatch cases were subjected to video monitoring, which might have resulted in more careful handling of the bycaught porpoises. Thus, this study might have underestimated the frequency of lesions related to disentanglement in bycaught animals. Besides, the presence of findings such as amputations and penetrating wounds or gaff marks could also mimic other types of trauma including those of other anthropogenic origin like collisions with ship propellers,^42^ or of a natural cause such as predatory attacks by grey seals (*Halichoerus grypus*).^34^

### Assessment of the General Health Status

Bycaught cetaceans are often considered as a better representation of the living population than stranded animals.^59^ Four of the 12 porpoises in this study had no other significant findings and could be considered “healthy” at the time of bycatch. Abnormalities were observed in the other cases, including 4 porpoises with severe or extensive disease or signs of debilitation that likely negatively affected these animals, making their chances of long-term survival questionable had they not been bycaught. Our study highlights the need to conduct systematic health assessments of incidentally caught cetaceans, as demonstrated previously.^30,31^

Bycaught porpoises in German waters presented poorer health status compared to those caught in Norwegian and Icelandic waters, but comparable health status to animals caught in Greenlandic waters.^59^ Ten percent of bycaught porpoises from the German Baltic Sea were emaciated.^59,60^ Parasite infestations in our cases were in line with previously reported loads, with infestation increasing with body size.^63^ Associated lesions were mild to moderate and were not considered as the sole direct cause of health problems, except for the one case where the parasite was found in the inner ear. The overall “low incidence of parasites” in our study is, however, likely biased by the fact that the majority of animals in this study were juveniles. Other studies describing pathological findings in bycaught harbor porpoises did not state significant pathological changes diagnosed as seen here. The proportion of bycaught porpoises in poor health is of concern and warrants further study of the broader population.

Two animals in this study were emaciated, and this was considered to be caused by inflammatory processes in multiple organs in one case, but undetermined for the other. The latter was the only case without any stomach content and, additionally, the only case in an advanced state of decomposition. It was deemed most likely that this animal was caught when already dead. Information regarding the fishing effort is vital in order to draw such conclusions. In this case, the gillnet was set for <12 h and the necropsy was performed the following morning. The state of decomposition of this animal reflects an animal that was dead for a longer time, at least exceeding 32 hours. Hence, occasional claims of fishermen that porpoises were already dead before they were bycaught^16^ may sometimes be true.

Food remains in the gastrointestinal tract were observed in all animals that died as a result of bycatch (*n* = 11). This represents a good indicator of an acute cause of death, like bycatch, although recent ingestion of prey is also seen in other acute causes of death including predatory attacks.^33^ Undigested prey species in stomachs of stranded animals that are suspected to have been bycaught could indicate the location of death, and prey species composition is therefore informative in regard to the manner of death.^35^ This is particularly true when fisheries in a certain geographic area target solely bottom or pelagic species. In the southern North Sea, bottom-set gillnets are the primary fishery type and demersal prey in stomachs of bycaught porpoises can therefore be expected. Although the majority of porpoises eat demersal prey in the southern North Sea, this is proportionally higher in the diet of porpoises dying in bottom-set gillnets as demonstrated previously.^35^ Additionally, stomach content analysis might also reveal clues about potential risk of bycatch. This was demonstrated in a study of bycaught Atlantic white-sided dolphins (*Lagenorhynchus acutus*) in pelagic trawlers, in which their diet was identified as the discarded fish from the trawlers, suggesting that this might have attracted the animals to the trawlers in the first place.^5^ Analyzing stomach contents as part of postmortem investigations is therefore recommended and would aid in the assessment and understanding of the bycatch problem. Besides gastrointestinal tract and prey species assessment, the presence of chyle in lymphatic ducts is also a reliable predictor of recent feeding^1^ and should be scored during future necropsies of stranded animals.

## Conclusion and Recommendation

The diagnosis of drowning relies on circumstances of discovery, inference, and the exclusion of other fatal conditions, because there are no pathognomonic lesions of drowning.^38,45^ Therefore, for deceased, stranded cetaceans, a range of tests and assessments is required in order to estimate the likelihood that death was caused by drowning following bycatch. The most common findings in the confirmed bycaught harbor porpoises available for this study were nonspecific lesions such as pulmonary emphysema, pulmonary alveolar edema, or organ congestion. More specific findings including incisions and net imprints were shown to be good indicators of bycatch. Recently ingested (demersal) prey also proved to be a good indicator, at least pointing toward an acute cause of death close to the sea floor. In contrast to previous studies, a favorable health status, the absence of disease, and good nutritional condition were shown to be poor indicators of bycatch, at least for porpoises caught in coastal waters of the southern North Sea. The proportion of stranded animals that result from bycatch may be underestimated if cases with notable pathological findings (eg, severe parasitism, poor nutritional condition, and infectious diseases) are excluded from the analysis. This demonstrates a need for understanding endemic disease and an appropriate calibration of diagnostic interpretations, taking into account a range of tests and investigations to establish the conclusion on the manner and most likely cause of death of stranded individuals.

Bycatch criteria gained from the literature review should be tested and validated when implemented in other geographical areas or fishery types. We stress the importance of obtaining more bycaught animals by cooperation with the fisheries sector for validation and further calibration of the results, because the number of landed harbor porpoises by fishermen was low. To understand the scale of the bycatch issue, monitoring programs are required.^56^ A good cooperation with the participating fishermen is needed to obtain bycaught marine mammals for postmortem investigations. Governmental support is required to acquire the necessary permits, and to meet the requirements of several international conventions aiming to reduce human-induced mortality levels. Last, based on the necropsy results of the animals included in this study, the health status of porpoises in the southern North Sea can, at a minimum, be considered of concern.

## Supplemental Material

Supplemental Material, Combined_supplemental_materials-IJsseldijk_et_al - Challenges in the Assessment of Bycatch: Postmortem Findings in Harbor Porpoises (*Phocoena phocoena*) Retrieved From Gillnets Click here for additional data file. Supplemental Material, Combined_supplemental_materials-IJsseldijk_et_al for Challenges in the Assessment of Bycatch: Postmortem Findings in Harbor Porpoises (*Phocoena phocoena*) Retrieved From Gillnets by Lonneke L. IJsseldijk, Meike Scheidat, Marije L. Siemensma, Bram Couperus, Mardik F. Leopold, Maria Morell, Andrea Gröne and Marja J. L. Kik in Veterinary Pathology
