# Supplementary material for: Challenges in the Assessment of Bycatch: Postmortem Findings in Harbor Porpoises (Phocoena phocoena) Retrieved From Gillnets
Source: Vet Pathol. 2020 Dec 4;58(2):405–15. doi: 10.1177/0300985820972454 (PMC7961740; doi:10.1177/0300985820972454)
Supplement: Supplemental Material, Combined_supplemental_materials-IJsseldijk_et_al - Challenges in the Assessment of Bycatch: Postmortem Findings in Harbor Porpoises (Phocoena phocoena) Retrieved From Gillnets [file Combined_supplemental_materials-IJsseldijk_et_al.pdf]

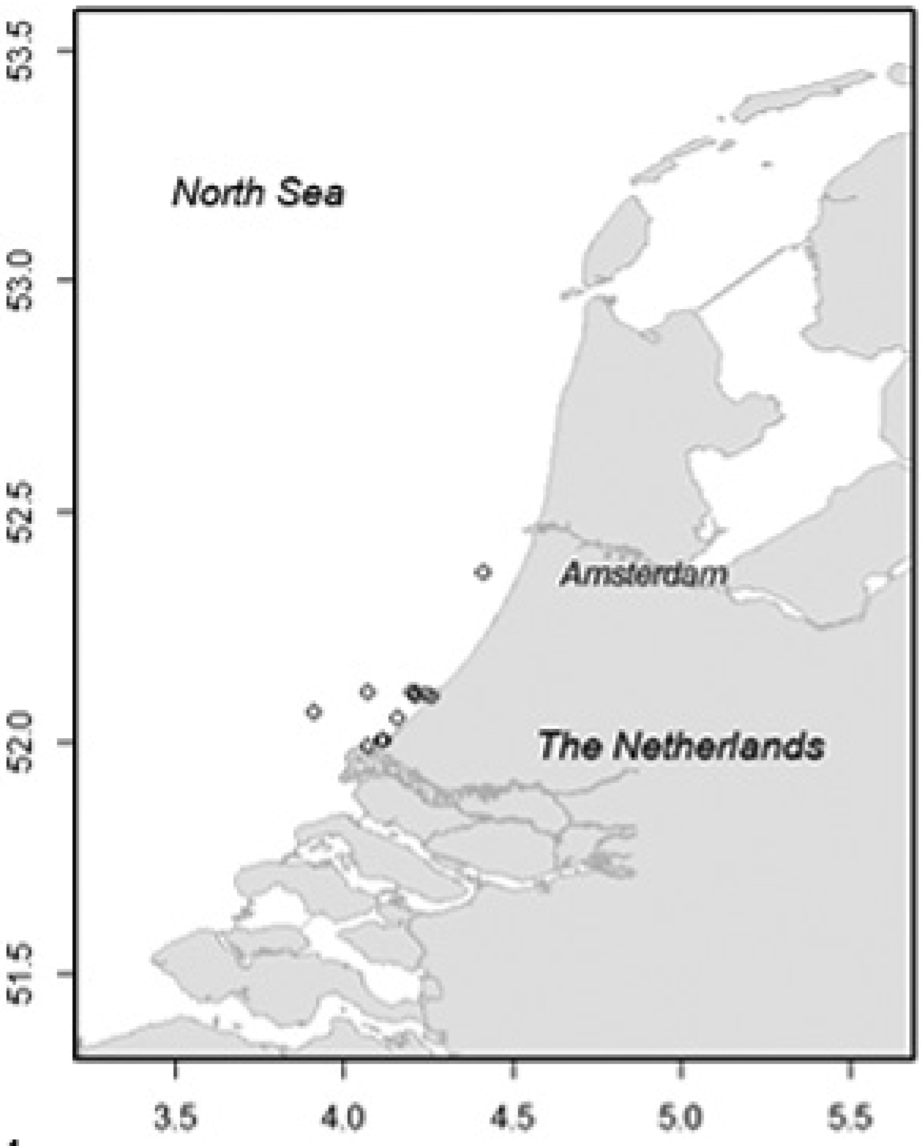

S1

Supplemental Figure S1. Locations of gillnets in which harbor porpoises included in this study (n=12) were bycaught.

Supplemental Table S1: Criteria gained from literature review with associated references

Criteria	Criteria reported by
1: Findings likely related to the drowning process	
Hyphema	Siebert et al. 2006; Bernaldo de Quirós et al. 2013
Pulmonary edema	Birkun 1994; García-Hartmann et al. 1994; Kuiken 1994; Lipscomb et al. 1994; Siebert et al. 1994; Knieriem & García-Hartmann 2001; García-Hartmann et al. 2004; Siebert et al. 2006; Jepson et al. 2013
Pulmonary emphysema	Birkun 1994; García-Hartmann et al. 1994; Kuiken 1994; Lipscomb et al. 1994; Siebert et al. 1994; García-Hartmann et al. 2004; Siebert et al. 2006; Jepson et al. 2013
Pleural petechiae or pulmonary hemorrhage	Birkun 1994; Kuiken 1994; Lipscomb et al. 1994; Knieriem & García-Hartmann 2001; García-Hartmann et al. 2004; Siebert et al. 2006
Changes in reticulum fiber structures of lung	Knieriem & García-Hartmann 2001
Regurgitation of food	Birkun 1994
Epicardial petechiae	Birkun 1994; Kuiken 1994; García-Hartmann et al. 2004
Organ congestion	Birkun 1994; Lipscomb et al. 1994; Bernaldo de Quirós et al. 2013; Jepson et al. 2013
Disseminated congestion	Birkun 1994; Bernaldo de Quirós et al. 2013
Presence of foreign material in lungs or bones	Birkun 1994; Larsen & Holm 1994
2: Findings related to the direct contact with the gillnet	
Superficial incisions in edges of the mouth, fins or tail(stock)/fluke	Birkun 1994; García-Hartmann et al. 1994; Kuiken 1994; Tregenza 1994; Siebert et al. 2001; García-Hartmann et al. 2004; Bernaldo de Quirós et al. 2013; Jepson et al. 2013
Encircling lesions, anywhere on the body	Tregenza 1994;
Subcutaneous hemorrhage/ contusions	García-Hartmann et al. 1994; Kuiken 1994; García-Hartmann et al. 2004; Jepson et al. 2013
Intramuscular hemorrhage, myofiber degeneration or skeletal muscle contusions	García-Hartmann et al. 2004; Siebert et al. 2006; Jepson et al. 2013
Disseminated gas bubbles	Moore et al. 2009; Bernaldo de Quirós et al. 2013
Pneumothorax	García-Hartmann et al. 1994
(Acute) skull or rib fractures	García-Hartmann et al. 1994; Kuiken 1994; Jepson et al. 2013
3: Findings related to disentanglement of bycaught animals	
Amputations	Birkun 1994; García-Hartmann et al. 1994; Kuiken 1994; García-Hartmann et al. 2004; Jepson et al. 2013
Presence of fishing material (netting, rope) around body extremities	Tregenza 1994; Kuiken 1994; García-Hartmann et al. 2004; Jepson et al. 2013
Gaff marks	Tregenza 1994; Kuiken 1994; García-Hartmann et al. 2004
Penetrating incision wounds or lacerations (tissue tears) into body cavities	Birkun 1994; Kuiken 1994; Tregenza 1994; García-Hartmann et al. 2004; Jepson et al. 2013
4: General health status of bycaught animals	
Good nutritional condition	Kuiken 1994; García-Hartmann et al. 2004; Siebert et al. 2006; Jepson et al. 2013
Low incidence of parasitism	García-Hartmann et al. 1994; Siebert et al. 2006
Exclusion of other cause of death	García-Hartmann et al. 1994; Kuiken 1994; García-Hartmann et al. 2004
Recently ingested gastric content	García-Hartmann et al. 1994; Kuiken 1994; Lipscomb et al. 1994; García-Hartmann et al. 2004; Bernaldo de Quirós et al. 2013; Jepson et al. 2013

Supplemental Table S2: Basic information of bycaught harbor porpoises

Case #	Date	DCC at retrieval	Frozen	Total length (cm)	Weight (kg)	Age class	Age (years)	Sex	DCC at necropsy	NCC
1	2008-02-13	1	Y	117	24.3	Juvenile	<1	Female	2	3
2	2011-01-31	1	N	115	31	Juvenile	<1	Male	1	1
3	2011-03-28	1	N	117	24.5	Juvenile	<1	Female	1	2
4	2013-09-04	1	N	141	38.5	Adult	10	Female	4	1
5	2013-12-10	1	N	101.5	16.2	Juvenile	<1	Male	1	6
6	2014-06-03	1	N	101.5	19	Juvenile	(<)1	Male	1	3
7	2014-10-06	1	N	93.5	13.7	Juvenile	<1	Male	1	4
8	2015-06-27	2	N	115	24.5	Juvenile	1	Male	2	2
9	2015-06-12	4	N	171	48	Adult	8	Female	4	6
10	2015-08-20	1	N	115	25.5	Juvenile	1	Female	1	3
11	2017-03-11	1	N	115	28	Juvenile	<1	Male	1	1
12	2019-05-27	2	N	115.5	24	Juvenile	1	Male	2	4

Veterinary Pathology: Supplemental Materials.
IJsseldijk et al. Challenges in the assessment of bycatch:
Post-mortem findings in harbor porpoises (*Phocoena phocoena*) retrieved from gillnets.

Supplemental Table S3: Overview of scored criteria and main pathological findings

Criteria	Based on	Case #1	Case #2	Case #3	Case #4	Case #5	Case #6	Case #7	Case #8	Case #9	Case #10	Case #11	Case #12
**Hyphema**	Gross assessment	Unilateral (L)	Bilateral	Absent	Bilateral	Unilateral (R)	Absent	Absent	Absent	Unknown	Unilateral (R)	Absent	Absent
**Amputations**	Gross assessment	No	No	No	No	No	No	No	No	No	No	No	No
**Presence of gear**	Gross assessment	No	No	No	No	No	No	No	No	No	No	No	No
**Penetrating incision/wound**	Gross assessment	No	No	No	No	No	No	No	No	No	No	No	No
**Gaff marks**	Gross assessment	No	No	No	No	No	No	No	No	No	No	No	No
**Subcutaneous hemorrhage**	Gross assessment	Present, submandibular, head and on M. longissimus dorsi	Present, on skull (caudaldorsally)	Present, mandible	No	No	No	No	Present, scapula	Likely not, but autolysis hampers diagnosis	No	No	No
**Intramusculature hemorrhage**	Gross assessment	No	No	No	No	No	No	No	No	Likely not, but autolysis hampers diagnosis	No	No	No
**Acute skull fractures**	Gross assessment	No	No	Yes, left mandible	No	No	No	No	No	No	No	No	No
**Gas bubbels**	Gross assessment	Unknown	No	No	Unknown	No	No	No	No	Unknown	No	No	No
**Pneumothorax**	Gross assessment	No	No	No	Unknown	No	No	No	No	Unknown	No	No	No
**Recent ingested gastric content**	Gross assessment	Present	Present	Present	Present	Some	Present	Present	Some	Absent	Present	Present	Present
**Regurgitation of food in upper alimentary tract**	Gross assessment	Absent	Absent	Present	Present	Absent	Present	Present	Absent	Absent	Absent	Absent	Absent
**Pulmonary edema**	Gross and histologic assessment	Present	Present	Present	Unknown	Present	Present	Present	Present	Unknown	Present	Present	Present
**Pulmonary emphysema**	Histologic assessment - HE	Absent	Present, associated to pneumonia	Present, associated to pneumonia and pleuritis	Unknown	Present, associated to pneumonia	Absent	Present, very little associated pneumonia	Present, associated to pneumonia	Unknown	Present, associated to pneumonia	Present, very little associated pneumonia	Present, associated to pneumonia
**Presence of foreign material in lungs**	Histologic assessment - HE	Unknown	No	No	Unknown	No	No	No	No	Unknown	No	No	No
**Petechiae - pleural**	Histologic assessment - HE	Present	Present	Present	Unknown	Absent	Absent	Absent	Absent	Unknown	Absent	Absent	Absent
**Petechiae - epicardial**	Histologic assessment - HE	Present	Absent	Present	Unknown	Absent	Absent	Absent	Absent	Unknown	Absent	Absent	Absent
**Skeletal muscle hemorrhage**	Histologic assessment - HE	Unknown	Absent	Absent	Unknown	Absent	Absent	Absent	Absent	Unknown	Absent	Absent	Absent
**Skeletal muscle myofiber degeneration**	Histologic assessment - PTAH	Unknown	Mild	Absent	Unknown	Absent	Unknown	Absent	Absent	Unknown	Absent	Absent	Mild
**Reticulum fiber structures lung**	Histologic assessment - HE and Gomori	Unknown	Unspecific	Unspecific	Unspecific	Unspecific	Unspecific	Unspecific	Unspecific	Unknown	Unspecific	Unspecific	Unspecific
**Organ congestion - adrenal**	Histologic assessment - HE	No	Present	Present	Unknown	Present	Present	Haemorrhage	Present	Unknown	Present	Present	No
**Organ congestion - brain**	Histologic assessment - HE	Unknown	Present	No	Unknown	No	Present	No	Present	Unknown	Present	Present	Present
**Organ congestion - heart**	Histologic assessment - HE	No	Present	No	Unknown	No	No	Present	Present	Unknown	Present	Present	No
**Organ congestion - kidney**	Histologic assessment - HE	No	Present	Present	Unknown	Present	No	Present	Present	Unknown	Present	Present	Present
**Organ congestion - liver**	Histologic assessment - HE	No	Present	No	Unknown	Present	No	Present	Present	Unknown	No	No	No
**Organ congestion - lung**	Histologic assessment - HE	Present	Present	Present	Unknown	Present	No	Present	Present	Unknown	Present	Present	Present
**Organ congestion - spleen**	Histologic assessment - HE	No	Present	No	Unknown	Present	No	Present	Unknown	Unknown	Present	Present	Present
**Multiple organ congestion - 2 or more**	Previous single organ assessment	Lung only	Yes, all 7	Yes 3	Unknown	Yes 5	Yes 2	Yes 5 or 6	Yes 6	Unknown	Yes 6	Yes 6	Yes 4
**Inner ear**	Scanning Electron Microscopy	Not conducted	Not conducted	Not conducted	Not conducted	Not conducted	Unilateral impaired hearing, focal hemorrhages in the cochlea	Not conducted	Not conducted	Not conducted	Focal hemorrhages in the cochlea	Not conducted	Not conducted
**Net marks, imprints or incisions**	Histologic assessment - HE	Focal loss of epidermis, some hemorrhage, some infiltrates	Focal loss of epidermis, no hemorrhage, no inflammatory reaction	Focal loss of epidermis, no hemorrhage, no inflammatory reaction	Not conducted	Focal loss of epidermis, some hemorrhage, no inflammatory reaction	Focal loss of epidermis, some hemorrhage, some infiltrates	Focal loss of epidermis, some hemorrhage, no inflammatory reaction	Focal loss of epidermis, no hemorrhage, no inflammatory reaction	Not conducted	Focal loss of epidermis, some hemorrhage, some infiltrates	Not conducted	Focal loss of epidermis, no hemorrhage, no inflammatory reaction
**Main pathological findings**	Gross and histologic assessment	No other significant findings, although freezing artefacts are apparent.	Myocarditis, multifocal mild enteritis, mild pneumonia, mild cholangioheptatitis	No other significant findings	Moderate verminous pneumonia. No histology	Chronic, multifocal, ulcerated and suppurative dermatitis, severe multifocal granulomatous bronchopneumonia with intralesional nematodes, ulcerated esophagus, chronic hepatitis and meningoencephalitis	Multifocal moderate bacterial dermatitis, subacute nephritis, mutlifocal severe verminous bronchopneumonia, mild diffuse enteritis, hepatitis, unilateral impaired hearing (Morell et al. 2017)	No other significant findings	Mild verminous pneumonia, mild hemochromatose	Severe parasitosis, moderate pneumonia. No histology	Focal severe proliferative dermatitis and multifocal mild viral dermatitis, moderate verminous and bacterial pneumonia	Mild encephalitis, mild verminous pneumonia	Osteomyelitis mandible (old trauma) and (bacterial) dermatitis (old trauma, most likely grey seal bites). Moderate verminous pneumonia.
**Comments**	All of above	No parafin embedded tissues available for reassessment/additional staining	NA	NA	Animal temporarily left in a harbor for the purpose of another study (findings not presented here), therefore DCC4 at necropsy and no histology taken.	NA	NA	NA	NA	Decomposed and most likely bycaught post-mortem	NA	NA	NA

Supplemental Table S4: Overview of net induced lesions and imprints observed during macroscopic assessment of bycaught harbor porpoises

	Pectoral fin(s)			Dorsal fin			Fluke			Mouth edges			Encircling imprint	
Case #	*Presence*	*Distribution*	*Severity*	*Presence*	*Distribution*	*Severity*	*Presence*	*Distribution*	*Severity*	*Presence*	*Distribution*	*Severity*	*Presence*	*Location*
1	Left and right side	Multifocal	Extensive	On trailing and leading edge	Multifocal	Mild	On trailing and leading edge	Multifocal	Extensive	Absent	NA	NA	Present	Cranial to pectoral fins around body
2	Right side	Multifocal	Severe	On trailing and leading edge	Focal	Moderate	On trailing and leading edge	Multifocal	Extensive	Absent	NA	NA	Absent	NA
3	Left and right side	Focal	Severe	On leading edge	Focal	Mild	On trailing and leading edge	Multifocal	Extensive	Present	Multifocal	Mild	Present	Around head
4	Left and right side	Multifocal	Extensive	On trailing and leading edge	Focal	Mild	On trailing and leading edge	Multifocal	Extensive	Present	Focal	Moderate	Absent	NA
5	Right side	Multifocal	Mild	Absent	NA	NA	Absent	NA	NA	Absent	NA	NA	Absent	NA
6	Right side	Multifocal	Mild	Absent	NA	NA	On trailing and leading edge	Multifocal	Moderate	Present	Multifocal	Severe	Present	On rostrum
7	Right side	Multifocal	Mild	On trailing edge	Focal	Mild	Absent	NA	NA	Present	Focal	Moderate	Present	On rostrum
8	Left and right side	Multifocal	Mild	On trailing and leading edge	Focal	Mild	On trailing and leading edge	Multifocal	Moderate	Absent	NA	NA	Present	On rostrum
9	Left and right side	Multifocal	Extensive	On leading edge	Focal	Mild	On trailing and leading edge	Multifocal	Extensive	Absent	NA	NA	Absent	NA
10	Left and right side	Multifocal	Extensive	On trailing edge	Focal	Mild	On trailing and leading edge	Multifocal	Mild	Present	Focal	Mild	Present	On rostrum
11	Left side	Focal	Severe	On trailing and leading edge	Multifocal	Mild	On trailing and leading edge	Multifocal	Extensive	Present	Focal	Mild	Present	On rostrum
12	Left and right side	Multifocal	Extensive	On trailing and leading edge	Multifocal	Extensive	On trailing and leading edge	Multifocal	Extensive	Present	Multifocal	Extensive	Present	On rostrum

Supplemental Table S5: Pelagic and demersal prey species, number and mass (in grams) per bycaught animal with stomach content (n=11) and total percentage of pelagic and demersal prey number and mass.

		Pelagic prey				Demersal prey							
Case #		*Lesser pipefish*	*Herring*	*Sprat*	*Smelt*	*Sandeel*	*Goby*	*Whiting*	*Five-beard*	*Bib*	Total	Total pelagic	Total demersal
1	*Number*	0	0	0	0	24	500	0	0	0	524	0	524
	Mass	0	0	0	0	212.58	239.4	0	0	0	451.98	0	451.98
2	*Number*	0	5	3	0	86	1585	0	0	0	1679	8	1671
	Mass	0	52.28	22.56	0	628.31	1383.77	0	0	0	2086.92	74.84	2012.08
3	*Number*	0	3	0	2	47	494	0	0	0	546	5	541
	Mass	0	34.46	0	4.29	293.37	407.76	0	0	0	739.88	38.75	701.13
4	*Number*	1	0	0	0	15	0	0	0	0	16	1	15
	Mass	0.24	0	0	0	261.14	0	0	0	0	261.38	0.24	261.14
5	*Number*	0	0	0	0	0	107	0	0	0	107	0	107
	Mass	0	0	0	0	0	132.22	0	0	0	132.22	0	132.22
6	*Number*	30	0	0	0	0	0	0	0	0	30	30	0
	Mass	14.96	0	0	0	0	0	0	0	0	14.96	14.96	0
7	*Number*	0	0	0	0	0	848	0	0	0	848	0	848
	Mass	0	0	0	0	0	958.54	0	0	0	958.54	0	958.54
8	*Number*	0	0	0	0	10	2	3	0	0	15	0	15
	Mass	0	0	0	0	89.79	0.30	222.56	0	0	312.65	0	312.65
10	*Number*	45	0	0	0	0	343	7	0	4	399	45	354
	Mass	25.04	0	0	0	0	404.39	427.03	0	112.03	968.49	25.04	943.45
11	*Number*	0	0	1	0	17	0	0	0	0	18	1	17
	Mass	0	0	23.11	0	79.28	0	0	0	0	102.39	23.11	79.28
12	*Number*	0	0	1	1	15	957	9	1	109	1093	2	1091
	Mass	0	0	10.94	15.41	52.9	2636.6	13.51	36.26	26.75	2792.37	26.35	2766.02

		Pelagic	Demersal	
92	5183	1.74	98.26	% -n
203.29	8618.49	2.30	97.70	% -M
